# Proliferation of *Listeria monocytogenes* L-form cells by formation of internal and external vesicles

**DOI:** 10.1038/ncomms13631

**Published:** 2016-11-23

**Authors:** Patrick Studer, Titu Staubli, Noémi Wieser, Patrick Wolf, Markus Schuppler, Martin J. Loessner

**Affiliations:** 1 Institute of Food, Nutrition and Health, ETH Zurich, Schmelzbergstrasse 7, Zurich CH 8092, Switzerland

## Abstract

L-forms are cell wall-deficient bacteria that divide through unusual mechanisms, involving dynamic perturbations of the cellular shape and generation of vesicles, independently of the cell-division protein FtsZ. Here we describe FtsZ-independent mechanisms, involving internal and external vesicles, by which *Listeria monocytogenes* L-forms proliferate. Using micromanipulation of single cells and vesicles, we show that small vesicles are formed by invagination within larger intracellular vesicles, receive cytoplasmic content, and represent viable progeny. In addition, the L-forms can reproduce by pearling, that is, generation of extracellular vesicles that remain transiently linked to their mother cell via elastic membranous tubes. Using photobleaching and fluorescence recovery, we demonstrate cytoplasmic continuity and transfer through these membranous tubes. Our findings indicate that L-forms' polyploidy and extended interconnectivity through membranous tubes contribute to the generation of viable progeny independently of dedicated division machinery, and further support L-forms as models for studies of potential multiplication mechanisms of hypothetical primitive cells.

## Abstract (web summary)

L-forms are cell wall-deficient bacteria that divide through unusual mechanisms, potentially resembling those of primitive cells. Here the authors describe how *Listeria monocytogenes* L-forms proliferate by generation of internal and external vesicles.

## Graphical abstract

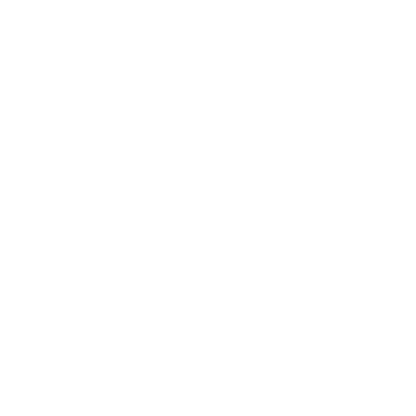

Bacteria are surrounded by a rigid cell wall that confers stability and defines the shape of the cell. Despite its pivotal role, bacteria are able to undergo a transition into a cell wall-deficient state, in which they adopt alternative lifestyles in order to survive and proliferate. These L-form bacteria were first discovered in 1935 (ref. 1), and have since been described for many Gram-positive and Gram-negative organisms. Generation of L-form bacteria is based on interference with peptidoglycan synthesis or integrity, followed by cultivation in osmotically adapted media^2^. The L-forms may assume a transient state (that is, reversion to the walled form is possible), or a stable state, in which they can be cultivated in absence of the inducing agent (without reversion). Several mutations were reported to support L-form conversion^3^,^4^,^5^, and it was also shown that deleterious mutations may accumulate during the L-form state, possibly preventing subsequent regeneration of a mature cell wall^6^.

Cell division usually is a tightly regulated process orchestrated by highly dedicated machinery, which guarantees precise distribution of components essential for autonomous life. Rod-shaped bacteria generally proliferate by binary fission, giving rise to two equal daughter cells. The tubulin homologue FtsZ represents a central player during this process and assembles a Z-ring at midcell, to which further division proteins are then recruited^7^,^8^. In contrast, L-form multiplication seems independent of FtsZ^3^,^9^ resulting in the evolution of various modes of proliferation, including binary fission, extrusion-resolution, blebbing, and extra- and intracellular budding^10^. The phenotypically different mechanisms can be explained by (or are the result of) an apparently unorganized and non-orchestrated proliferation^4^,^10^,^11^. Increased membrane synthesis and changes in the surface-to-volume ratio might be sufficient to drive enlargement and vesicular proliferation in absence of other components^3^,^4^,^9^,^12^,^13^. The apparent ability of L-forms to replicate without cell division machinery suggests L-forms as attractive model for top-down studies on the properties of hypothetical primitive cells^10^,^14^. However, as an apparent consequence of uncoordinated proliferation and uneven distribution of essential elements such as a chromosome, ribosomes and a basic set of metabolic enzymes, only a fraction of L-form progeny seem to be viable.

Here we study the different proliferation modes of a stable L-form of *Listeria monocytogenes*. Time-lapse imaging of growing cells reveals highly variable shape perturbations that result in FtsZ-independent generation of both intra- and extracellular daughter vesicles. Micromanipulation for selective isolation of single cells and internal vesicles enables us to refine our previous hypothesis regarding viable reproduction units^13^,^15^. In particular, our findings indicate that primary internal vesicles (PIVs) within the L-form do not represent viable offspring. Rather, the much smaller secondary internal vesicles (SIVs, formed inside the PIVs) are viable progeny. Newly formed L-forms in liquid culture remain transiently linked to each other, via filament-like tubular strands of lipid material. These strands show a high degree of mechanical flexibility and stability, mediate cytoplasmic continuity and allow transfer of cytoplasmic material between cells. Therefore, the highly polyploid nature of L-forms, and the transfer of essential molecules through membranous tubes, contribute to the generation of viable progeny in the absence of dedicated division machinery.

## Results

### *Listeria* L-forms produce intra- and extracellular progeny

**Figure 1:**
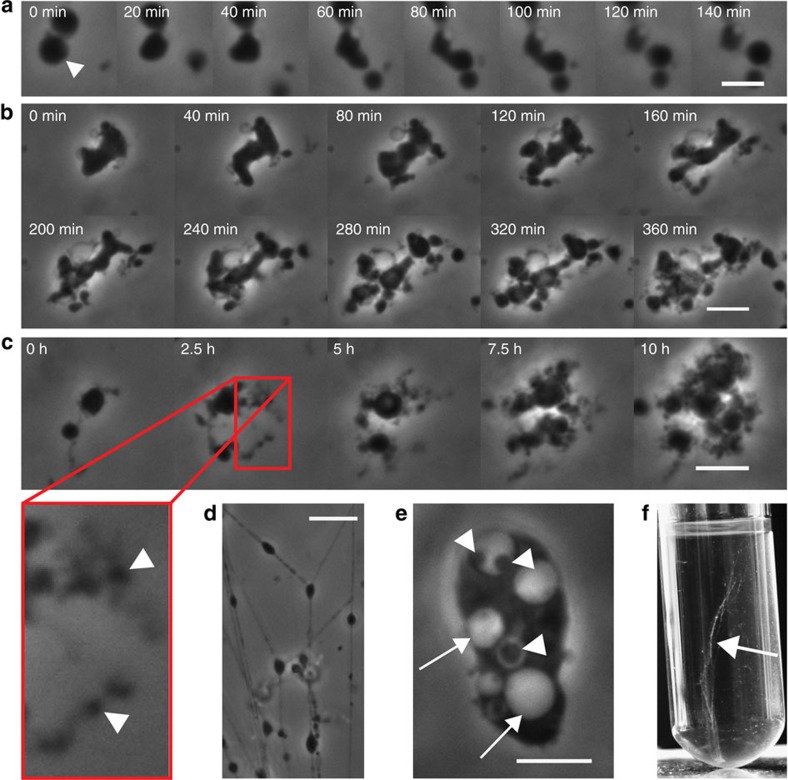
Uncoordinated proliferation in *Listeria* L-forms. (**a**) Generation of a new L-form by a budding-like process (indicated by arrowhead). (**b**) L-forms often generated multiple protrusions that eventually fragmented, thereby producing new cells. (**c**) Two cells that were connected by a thin strand of presumably membrane-like lipid material. Over a period of 10 h, a massive production of strand material and associated new extracellular vesicles (EVs, arrowheads in enlargement) was observed. (**d**) Growing L-forms in liquid medium often remained connected to each other. (**e**) A cell featuring secondary internal vesicles (SIVs; arrowheads). The content of the SIVs have a similar phase contrast intensity as the mother cell cytoplasm, whereas the content of the primary vesicles (PIVs, arrows) is similar to the surrounding medium. (**f**) The strand-like nature of L-forms during exponential growth can be observed by eye in static liquid culture, where bundles of L-forms produce structures that span the whole culture tube (arrow). Scale bars, 4 μm for (**a**), 8 μm for (**b**–**e**).

In earlier studies on proliferation^13^,^15^ and pathogenicity^16^ of *L. monocytogenes* L-forms, we used strains that were only able to grow in soft agar media. Here, we employed a stable L-form derivative of *L. monocytogenes* strain EGDe able to grow in various media, including liquid culture, soft agar and agar plates^17^. To investigate in more detail how these cells proliferate under different conditions, we established an L-form live cell imaging platform. L-forms grown in liquid culture were transferred into multi-well glass bottom dishes, carefully centrifuged and spun onto the glass layer, and overlaid with *Listeria* L-form medium (LLM) soft agar to minimize passive motility, that is, Brownian motion of the cells. Employing a temperature-controlled incubation chamber assembled on the confocal microscope stage, L-form growth and multiplication was observed and recorded over extended time periods. Overall, cell proliferation was found to be quite heterogeneous, and several distinct proliferation modes were observed. In a first step, the initially spherical L-form (mother or donor) cells adopted a more irregular shape, before they started to generate protrusions that eventually led to generation of new (daughter or recipient) cells. These shape perturbations appeared to be membrane-synthesis dependent, and often resembled a budding-like process (Fig. 1a, Supplementary Movie 1). In other instances, cells formed multiple evaginations that almost simultaneously pinched off, resulting in production of several progeny cells at once (Fig. 1b, Supplementary Movie 2). Interestingly, newly formed extracellular vesicles (EVs) tended to remain connected to the mother cell by thin strands of presumably membrane-like material (Figs 1c,d [Fig f2] [Fig f3] [Fig f4]and 5 and Supplementary Fig. 1). These strands often featured small EVs (a phenotype known as ‘pearling'^10^), which also represent potential progeny (Fig. 1c, arrowhead). Interestingly, the occurrence of such strands or similar structures had also been reported for L-forms of other genera, including *Streptobacillus*^1^ and *Bacillus*^18^, but their function remained unknown. Employing extended time-lapse microscopy of *Listeria* L-forms over periods of up to 10 h enabled us to monitor differentiation of the membrane strands into cell-like elements (Fig. 1c, Supplementary Movie 3). Interestingly, the strand-like morphology of L-form cells could also be observed by eye in liquid culture, where the visible growth frequently formed long, thread-like assemblies (Fig. 1f, arrow). Larger, matured L-form cells often contained primary intracellular vesicles (PIVs, Fig. 1e arrows), which can harbour one or more smaller secondary intracellular vesicles (SIVs, Fig. 1e, arrowheads). Appearance of the PIVs in phase contrast microscopy frequently appeared similar to the medium, suggesting that they may actually contain extracellular fluid (Fig. 1e, arrow). In contrast, the SIVs generally appeared much denser and similar to the cytoplasmic material of the mother cell, which suggested that they contain cytoplasm transferred from the mother cell. Although emergence of the PIVs and especially the SIVs was difficult to follow by time-lapse microscopy, it was evident that they were very small upon first appearance inside the cell, but subsequently increased in size along the growing mother cell (Supplementary Movie 4). Interestingly, there seems to be a limitation for L-form multiplication, that is, individual cells stopped producing new vesicles at some point, but continued to increase in size (including PIVs and SIVs) for several hours.

### L-form proliferation is independent of FtsZ

**Figure 2 f2:**
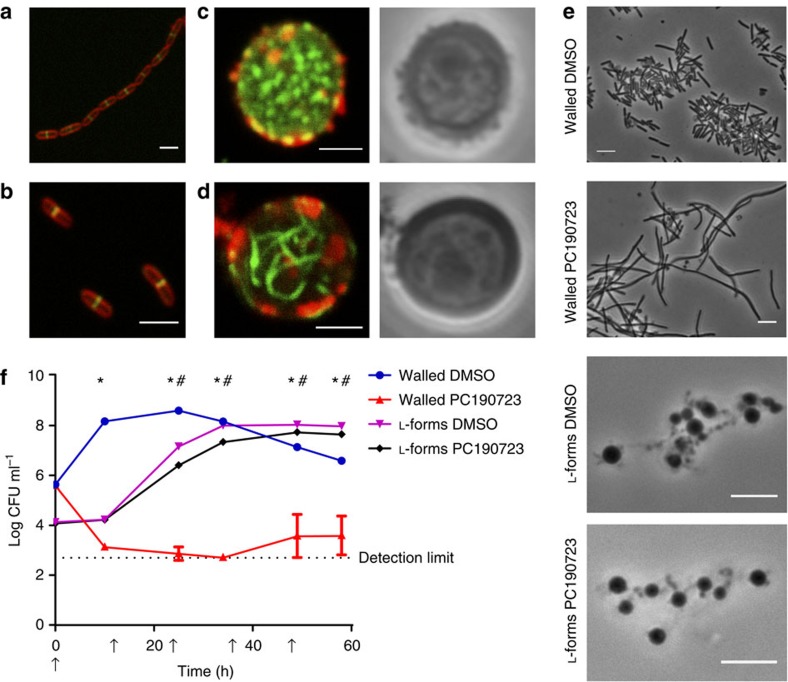
FtsZ is dispensable for L-form proliferation. (**a**) FtsZ-GFP (green) expressed from a multicopy plasmid leads to a chain formation phenotype in walled cells, and localizes to the septal region. FM 4–64 (red) was used as lipid counterstain. (**b**) Lower expression levels of FtsZ-GFP (green) from a single copy, integrative plasmid abolished the chain formation phenotype in walled cells, while maintaining the septal localization of FtsZ-GFP. FM 4–64 (red) was used as lipid counterstain to visualize cell boundaries. (**c**) In an L-form, FtsZ-GFP (green) generally formed short filaments, resulting in a spotty pattern throughout the cell. Membranes were stained red with BTME. (**d**) In some cases, unorganized, longer FtsZ-GFP filaments (green) were observed in L-forms. Membranes were stained red with BTME. (**e**) Walled *L. monocytogenes* grown in presence of the FtsZ inhibitor PC190723 formed long, filamentous cells, whereas L-forms did not show morphological aberrations in presence of the inhibitor. (**f**) Growth of parental cells in presence of the FtsZ inhibitor PC190723 was abolished, while L-forms continued to grow in presence and absence of PC190723. The inhibitor was supplemented in intervals of 12 h (arrows) to prevent its depletion by inactivation. Values represent average±s.d. of three independent cultures (*n*=3). Asterisks indicate *P*<0.05 for walled samples based on an unpaired *t* test. Hash marks indicate *P*<0.05 for L-form samples based on an unpaired *t* test. Scale bars, 2 μm for (**a**–**d**), 5 μm for (**e**).

As noted above, the shape perturbations occurring during L-form proliferation appeared very heterogeneous and uncontrolled. In addition, it was shown for L-forms of other genera that they can divide independent of FtsZ^3^,^9^. Therefore, we addressed the questions whether *Listeria* L-forms continue to employ the FtsZ-based cell division machinery used by normal rod-shaped walled cells. First, we determined FtsZ localization employing a mutant strain featuring an ectopic *ftsZ-gfp* fusion expressed under control of an inducible promoter^19^ on a multicopy shuttle vector^20^. Although it is known that GFP tagged FtsZ might interfere with its function and ultimately the division process if the expression level is higher than 25% of the native FtsZ^7^, we observed a septal location of the protein, as reported previously^21^ (Fig. 2a). To determine if the somewhat irregular chain-forming phenotype of the cells was due to too high levels of the GFP fusion, we inserted the gene fusion into the single-copy integration vector pPL2 (ref. 22). As a result, mutant cells displayed correct localization of FtsZ, but without chain formation (Fig. 2b). When the L-form cells were transformed with these plasmids, we found no abnormal morphology irrespective of the expression levels of FtsZ-GFP, which suggested that FtsZ might have lost its function. Interestingly, two distinct FtsZ-GFP localization patterns were observed in L-forms: in the majority of cells, FtsZ-GFP formed many short filaments which results in a spotty pattern throughout the cells (Fig. 2c). In some cases, however, long filaments were formed (Fig. 2d) that spanned the entire cytoplasm. For better visibility, images are presented as 3D reconstruction of Z-stacks (Supplementary Movie 5).

We wanted to determine whether L-forms could multiply in absence of functional FtsZ, which organizes cell-division^7^, and used PC190723 as specific inhibitor^23^. The drug blocks the inherent GTPase activity of FtsZ, and stabilizes the otherwise highly dynamic FtsZ filaments in a dose-dependent manner^24^. Cell counts of walled *Listeria* cells cultivated in presence of the compound decreased drastically (Supplementary Fig. 2a), whereas L-forms continued to grow at the same or even four-fold higher concentration of the drug (Supplementary Fig. 2b,c). Yet, the initially inhibited walled cells resumed growth after prolonged incubation periods, possibly due to degradation of the inhibitor (Supplementary Fig. 2a). In follow-up experiments, we used higher concentrations of PC190723 (16 μg ml^−1^ instead of 8 μg ml^−1^), and added the drug at 0, 12, 24, 36 and 48 h. As a result, walled cells suffered from a severe growth defect, whereas the L-forms continued to grow with and without repeated addition of the inhibitor, albeit at a slightly lower growth rate (Fig. 2f). Microscopical observations of walled cells revealed a filamentous morphology indicating absence of a functional Z-ring and lack of septum formation, whereas the morphology of L-forms cells did not reveal obvious changes (Fig. 2e). In order to verify that PC190723 stabilizes FtsZ filaments in L-forms and thus inhibits its function, we studied its effect on FtsZ-GFP spatial distribution. In treated cells, the majority of FtsZ-GFP should appear in the polymerized form, which can be visually differentiated from monomeric FtsZ-GFP by stronger fluorescence based on close spatial accumulation in the filaments. Accordingly, we found that polymerized FtsZ-GFP dominated in L-forms grown in presence of the inhibitor, whereas the untreated cells showed a mixture of polymerized and free FtsZ-GFP (Supplementary Fig. 3a). Quantification of the normalized histograms of ten cells grown in presence or absence of the inhibitor confirmed this observation (Supplementary Fig. 3b). Altogether, these findings confirm that PC190723 acts on FtsZ present in both rod-shaped and L-form cells, and all evidence strongly suggests that functional FtsZ and its associated machinery is dispensable for proliferation of wall-deficient L-form cells.

### Secondary internal vesicles represent viable progeny

**Figure 3 f3:**
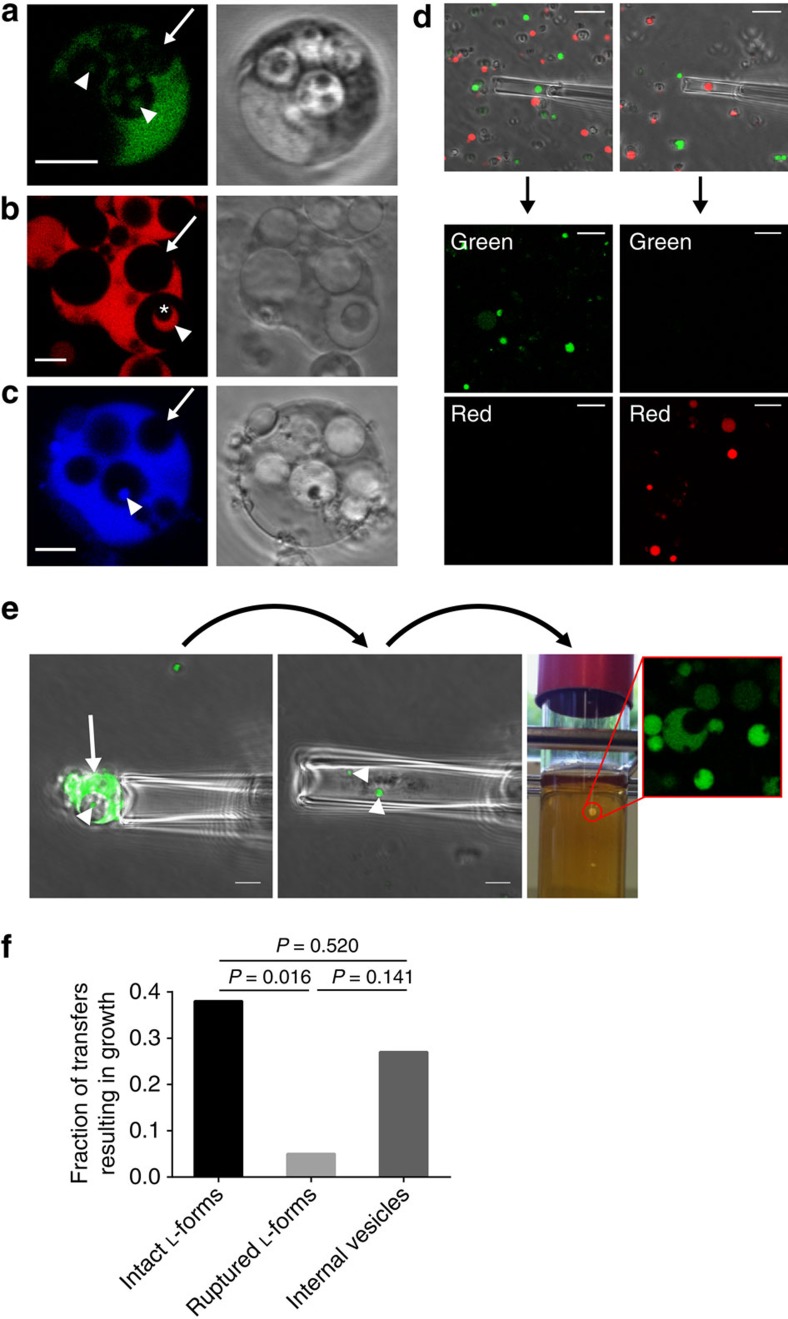
SIVs represent viable units. (**a**–**c**) SIVs (arrowheads), but not PIVs (arrows), contain cytoplasmic content of the surrounding mother cell. Confocal microscopy revealed the presence of GFP (**a**) and RFP (**b**) produced by the L-forms. Presence of DNA in SIVs was indicated by staining with Hoechst 33342 (**c**). SIVs may harbour tertiary intracellular vesicles (TIVs), which however did not contain cytoplasmic content (asterisk in **b**). (**d**–**f**) Micromanipulation was used as a tool to determine viability of isolated internal vesicles. (**d**) GFP or RFP expressing cells were isolated out of a mixture of both cells to prove feasibility of the approach to isolate single cells. (**e**) Representative image series of how internal vesicles were isolated and observed for their capability to form a colony. (**f**) Fraction of transfers resulting in growth. *n*=29, 20, 15 for ‘Intact L-forms', ‘Ruptured L-forms' and ‘Internal vesicles', respectively. *P* values calculated with the Fisher's exact test are depicted. Scale bars, 4 μm for (**a**–**c**), 5 μm for (**d**,**e**).

As described above, within the relatively large PIVs, small SIVs were frequently observed, which seem to contain cytoplasmic material likely originating from the mother cell (Fig. 1e, arrowheads). As a consequence, SIVs harbouring mother cell cytoplasm including essential components such as a chromosome, ribosomes and metabolic enzymes may be viable entities. To test this hypothesis, we wanted to show intracellular transfer of cytoplasmic material into vesicles. For this purpose, L-form strains featuring cytoplasmic reporters were generated, employing a PEG-based plasmid transformation protocol^25^ adapted for *L. monocytogenes* L-form cells. We created two different strains, expressing green (GFP) or red (RFP) fluorescent proteins. Confocal fluorescence microscopy confirmed the presence of corresponding markers inside the SIVs (Fig. 3a,b, arrowheads), and their absence in the larger primary vesicles (Fig. 3a,b, arrows). Staining of nucleic acids indicated the presence of chromosomal DNA within the smaller SIVs (Fig. 3c, arrowhead), but not the larger PIVs (Fig. 3c, arrow). Interestingly, in some cases we observed SIVs which themselves seemed to contain even smaller tertiary internal vesicles (TIVs), but these were non-fluorescent (Fig. 3b, asterisk). The TIVs likely also emerge from invagination of membrane into the SIVs, and therefore receive the non-fluorescent content from the PIVs. This observation further supports our conclusion that the SIVs receive cytoplasmic content from the original L-form mother cell cytoplasm.

Although these results already suggested that SIVs actually represent viable reproduction units, the ultimate proof was to be able to independently culture isolated SIVs. Towards this aim, micromanipulation was employed as a tool for isolation of SIVs directly from L-form cells. To test and demonstrate applicability of this approach, we adopted a microcapillary technique for isolation of single GFP or RFP expressing L-form cells from mixed culture of both strains, followed by transfer into fresh media and on agar plates. Inspection of colonies revealed pure communities of either GFP or RFP expressing cells (Fig. 3d). As a next step, single L-form cells (Fig. 3e, arrow) containing larger SIVs (Fig. 3e, arrowhead) were aspirated into the front end of a 6 μm diameter glass capillary. Then, the internal vesicles were released into the capillary, by mechanical disruption of the mother cell membrane with the aid of a second 0.4 μm diameter ultra-microcapillary. The liberated vesicles could then be transferred into fresh soft agar medium. Extended incubation for up to 16 days resulted in the appearance of L-form colonies (Fig. 3e, red circle) in 27% (*n*=15) of the transfers (Fig. 3f). GFP fluorescence served as control for origin from the initial culture (Fig. 3e). It should be noted that the above-mentioned transfer of intact L-form cells also yielded colonies in only 38% (*n*=29) of the transfers (Fig. 3f), which suggests a rather inefficient proliferation mode of L-forms. As control, the cellular contents of L-forms apparently devoid of SIVs were also isolated and cultured in the same way. We found that only 5% (*n*=20) of the transfers yielded viable L-forms (Fig. 3f), and this was likely due to very small SIVs not recognized by microscopical screening and selection. In conclusion, the findings support that the secondary vesicles receive cytoplasmic content from the mother (donor) cells and represent viable reproduction units.

### L-form cells are highly polyploid

**Figure 4 f4:**
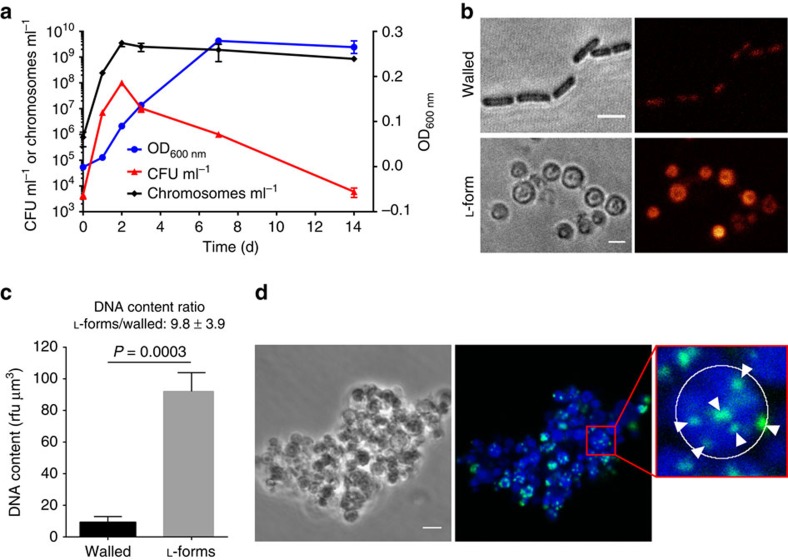
Growth kinetics and polyploidy of *Listeria* L-forms. (**a**) Growth was quantified by three different methods in parallel: optical density measurement, CFU determination by plating and chromosome quantification by qPCR. The discrepancy between the numbers for CFU ml^−1^ and chromosomes ml^−1^ is a measure for the polyploidy of L-forms. Note that the *y* axis for CFU ml^−1^ and chromosomes ml^−1^ is in a logarithmic scale, whereas the *y* axis for OD_600nm_ is in a linear scale. Values represent average±s.d. of three independent cultures (*n*=3). (**b**,**c**) Quantification of the Hoechst 33342 signal (red-orange pseudocoloured) in walled and L-form cells (**b**) revealed a ten-fold higher DNA content in L-forms than in parent cells (**c**). The quantification was performed as described in Supplementary Fig. 4. The bars represent average±s.d. of three independent cultures per cell type (*n*=3). The Hoechst 33342 signals of at least 50 cells per culture were determined. The *P* value of an unpaired *t* test is indicated. (**d**) Growth of L-forms exposed to the thymidine analogue EdU for 1 h and subsequent labelling with Alexa Fluor 488 (green) shows L-form cells featuring multiple DNA replication sites. The enlargement shows an L-form with at least six replication sites (arrowheads). The white circle depicts the cell boundaries based on the phase contrast channel. Hoechst 33342 staining (blue) was used to visualize the total DNA content of L-forms. Scale bars, 2 μm for (**b**), 4 μm for (**d**).

To properly quantitate L-form growth in liquid media, we employed three different methods. In addition to optical density measurement and CFU determination by plating, chromosome copies were determined in parallel using qPCR (Fig. 4a). Our findings confirmed previous results obtained for L-form growth on semi-solid media, that is, that L-form cells grew very slow and reach stationary phase after 2 days of incubation, and viable counts slowly decrease thereafter. In addition, quantitative determination of chromosome copies revealed that genome replication ceased after reaching the stationary phase plateau. In contrast, optical density continued to increase until day 7, and remained constant thereafter. This finding supports the observations from time lapse microscopy, that is, that mature L-forms continue to metabolize and increase in size, even though multiplication had stopped.

Taken together, the obvious discrepancy between viable counts and chromosome copies indicates the highly polyploid nature of these *Listeria* L-forms^3^,^26^. The average chromosome copy number for L-forms of *L. monocytogenes* strain ScottA was reported to be 18.0±3.6 (ref. 15), while the EGDe L-forms studied here contained approximately 34.5±5.8 chromosome copies per colony-forming unit (that is, viable cell), during exponential growth in liquid culture. However, considering that only approximately one-third of the L-form cells were able to form a colony (Fig. 3f), the actual chromosome number per cell is much lower, corresponding to approximately 13 copies per cell.

In another approach to determine polyploidy of L-forms and to verify the results described above, we used the DNA intercalating dye Hoechst 33342 in L-form cells in combination with quantitation of the fluorescence signals (Fig. 4b). The imaging analysis (described in Supplementary Fig. 4) revealed an approximately ten-fold higher DNA content in growing L-forms compared to rod-shaped cells (Fig. 4c). Assuming an average of 1.5 chromosomes per rod, an L-form cell contains approx. 15 chromosome copies, which is within the same range as indicated by the other methods. To provide even further support, multiple active chromosomal replication sites were observed in L-form cells grown in presence of the modified thymidine analogue EdU, followed by fluorescent labelling (Fig. 4d).

### Lipidic filaments enable transfer of cytoplasmic material

**Figure 5:**
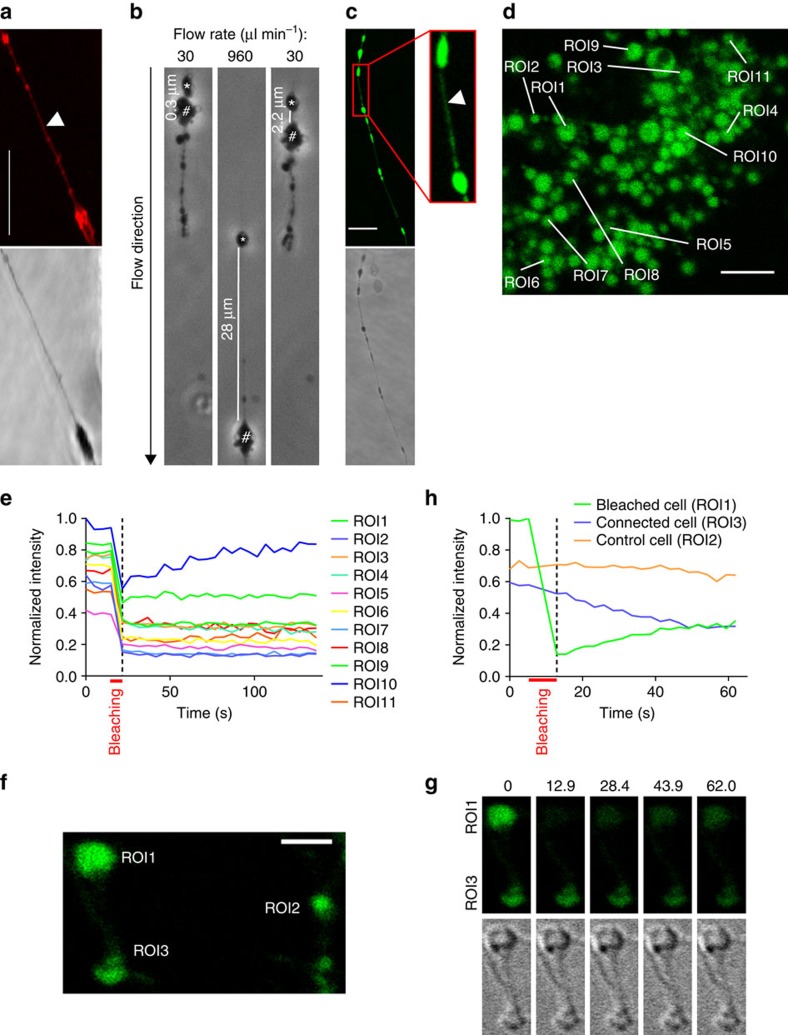
Filamentous lipid strands connecting newly formed EVs feature high mechanic stability and allow exchange of cytoplasmic content. (**a**) Staining with the lipid dye FM 4–64 reveals the membrane origin of the strands. (**b**) Inside a microfluidic flow cell, strands connecting separate cellular entities were stretched by exposure to increasing flow rates (shear stress), and immediately retract upon lowering the flow rate, without rupture. The asterisks and hashtags mark the identical cells in every image. The images were extracted as stills from a movie recorded during microscopy (Supplementary Movie 6). (**c**) Connecting strands contain GFP (green) produced by viable L-form cells. (**d**,**e**) Simultaneous bleaching of multiple L-forms within a microcolony revealed fluorescence recovery in one (ROI10) of the bleached cells. (**f**–**h**) Fluorescence loss in photobleaching (FLIP) was performed on different cells connected by a visible strand. (**g**,**h**) Bleaching of one of the two connected cells (ROI1) resulted in a more than 80% decrease in fluorescence signal intensity. Subsequently, however, the intensity of the unbleached partner cell (ROI3) gradually decreased (dashed line), whereas the intensity of the bleached cell (ROI1) gradually increased, indicating exchange of GFP between the connected compartments. Fluorescence intensity of an unconnected single cell (ROI2) remained constant over the duration of the experiment. (**g**) Selected time points (in s) during fluorescence recovery in ROI1 (upper cell) connected to ROI3 (lower cell). The DIC panel below shows that the cells did not move or change focal plane during the experiment. For an assembly of all timeframes, see Supplementary Fig. 6. Scale bars 8 μm for (**a**,**c**), 5 μm for (**d**), 2 μm for (**f**).

During growth in liquid culture, multiplying L-form cells frequently appeared to remain connected to each other via thin filament-like strands, presumably consisting of membrane material (Fig. 1c,d,f). The lipid-based nature of the strands was confirmed by staining with FM 4–64 dye (Fig. 5a). Hoechst 33342 staining revealed the presence of DNA in some of the strands (Supplementary Fig. 5a), whereas others were devoid of any detectable DNA (Supplementary Fig. 5b). We assumed that the filaments may break and release individual cells upon physical shear stresses such as mixing and swirling of the cultures. To assess rigidity of the lipidic strands, we exposed growing L-form cells to controlled shear stress, by immobilization in a miniaturized flow-cell chamber and application of defined flow rates of the medium. Figure 5b shows two L-form cells connected by a strand of approximately 0.3 μm in length, at an initially low flow rate of 30 μl min^−1^. By increasing the flow rate to 960 μl min^−1^, this strand could be stretched to 28 μm length (almost 100-fold) without rupturing it, and it reversed back to 2.2 μm length after the flow rate was decreased again (Supplementary Movie 6). This observation demonstrated the stability and flexibility of the connecting intercellular lipid filaments.

We next determined whether the lipidic strands may possibly represent a connection channel between the cytoplasm of adjacent cells, and if cytoplasmic content could be transferred through the strands. Such a mechanism would have important implications for how progeny cells would receive essential components. Microscopic examination of the connecting strands revealed that they are filled with cytoplasmic GFP originating from the L-form cells (Fig. 5c). To determine if an exchange of cytoplasmic material through these apparently tubular strands can occur between connected cells, we used ‘Fluorescence Loss in Photobleaching' (FLIP) experiments to follow the fate and replenishment of cytoplasmic GFP. In case the cytoplasm of different cells would remain connected, the bleached (non-fluorescent) GFP within the treated cell could be substituted by intact GFP from the associated cell. As a consequence, fluorescence should recover in the bleached cell, and proportionally decrease in the connected cell. For this, we first monitored fluorescence intensity in multiple cells within a colony, before and after bleaching. Some cells showed partial fluorescence recovery, yielding first evidence that cytoplasmic exchange is possible in L-forms (for example, regions of interest (ROI) 10 in Fig. 5d,e). Then, in a defined approach, single cells connected by strands were identified and specifically laser-bleached, while the fluorescence was continuously monitored in both cells. In some cases, recovery of fluorescence by transfer of GFP from a fluorescent cell into a bleached one was observed. As shown in Fig. 5f–h, the fluorescence intensity of a bleached cell (ROI1) dropped by more than 80% after treatment, but started to recover immediately after bleaching stopped. Consequently, fluorescence of the connected cell (ROI3) started to decrease, obviously due to the transfer of intact GFP molecules into the receiving cell and concomitant loss of its own cytoplasmic GFP pool. As a control, fluorescence intensity of a control cell (ROI2) did not change, demonstrating stability of the setup. Another example of laser bleaching of one cell and subsequent concomitant loss of fluorescence in a connected cell is shown in Supplementary Movie 7 and Supplementary Fig. 7. Altogether, these results indicate that the membranous tubes in fact allow cytoplasmic continuity and transfer of cytoplasmic components between connected cells.

## Discussion

Recent findings suggest that L-forms do not only differ morphologically from their walled counterparts, but may also feature changes on the genetic and molecular levels. This is especially evident with respect to cell division, which seems to be based mostly on biophysical processes^4^,^9^, and does not require certain cell division factors^3^,^12^.

With the aim to better understand *Listeria* L-form proliferation independent of the ‘classical' cell division machinery (Fig. 2), we employed various approaches to study the different, yet uncoordinated mechanisms used by these cell wall-deficient bacteria (Fig. 1). Because of the unorganized and inefficient nature of L-form multiplication, it was not surprising to find that only approximately one third of the newly formed offspring cells were viable and able to form a colony (Fig. 3f). For internal vesicles, the ratio was even lower (Fig. 3f), which could be explained by their small size and volume, which lowers the probability of acquiring a complete genome copy and all other essential cytoplasmic components during vesicle formation alone. Interestingly, our findings are consistent with the results from a study more than 40 years ago focusing on the viability of small internal ‘granules' in putative *Streptococcus* L-forms^27^. These L-forms featured far more granules inside their large bodies than CFU were produced following plating, indicating that not all granules were viable, which agrees very well with the findings reported here.

In our study, live cell imaging of *L. monocytogenes* L-form growth in liquid culture revealed that new L-form cells remain connected to each other by ultrathin tubes or strands consisting of lipidic membrane material. We speculate that the high stability and extreme elasticity of the strands may be based on the physical properties of chromosomal DNA present inside the strands, which could potentially stabilize the structures. However, we also observed strands without internal DNA (Supplementary Fig. 5b). Moreover, it has been shown that similar structures can be formed from artificial nucleic acid-free lipid vesicles^28^,^29^. Thus, internal DNA might play a role in the high elasticity of the strands, but the formation of the strands does not require presence of internal DNA.

The direct link between donor and recipient cells likely plays an important role in efficient L-form multiplication, since it allows the exchange of essential components between connected compartments. Therefore, it is possible that newly generated lipid vesicles can acquire essential components at a subsequent time point, as long as the cells remain connected. This process most likely involves passive distribution of essential molecules into the newly formed vesicles, which should significantly enhance the ratio of viable L-form generation over non-viable membrane vesicles. However, entire chromosomes may possibly be too large for transmission through the small channels formed by the connecting strands. This disruption of the close link between genome replication and cellular division in regular cells results in an unusual accumulation of genetic material in the L-form cells^3^,^26^. Thus, genome distribution to newly produced vesicles may simply occur passively by chance; if this is so, then the fraction of viable cells may increase with the density of chromosomes in the mother cell cytoplasm.

Uncoordinated multiplication of L-form bacteria has also been reported in other studies, which resulted in different, partially contradictory models for L-form proliferation, including binary fission, extrusion-resolution, extra- and intracellular budding^10^. These discrepancies may be due to various reasons, including the use of different organisms, growth media and induction protocols, and the very heterogeneous and variable properties of L-forms. As an example, the high degree of interconnectivity observed in strand-like growth of *Listeria* L-forms appears most pronounced during exponential growth, and declines with the age of the culture. In contrast, the number of L-forms containing intracellular vesicles (PIVs and SIVs) increases with the age of the culture.

**Figure 6:**
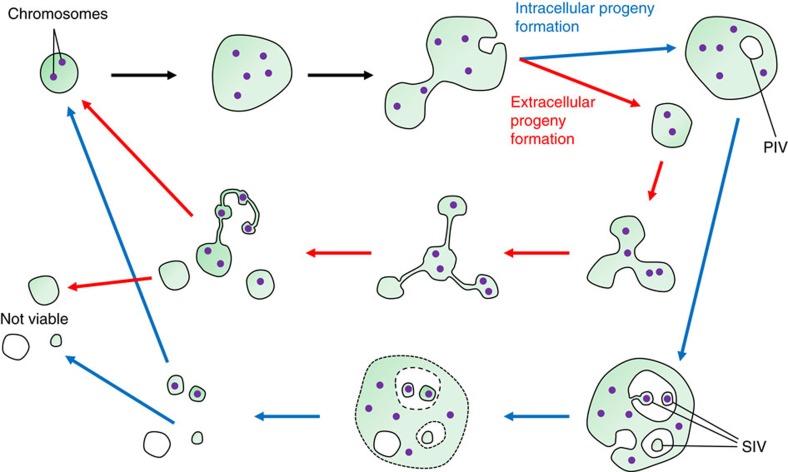
Revised model of *Listeria* L-form proliferation mechanisms. As a prerequisite, only those cells receiving all essential cytoplasmic components, that is, chromosomes (purple dots), ribosomes, enzymes, may become viable progeny. The initially round L-form cells (top left) may gradually transform into a more irregular shape, due to excess membrane lipid synthesis. This results in numerous invaginations and evaginations that may eventually pinch off, resulting in EVs containing mother cell cytoplasm, or PIVs containing extracellular fluid. Further evaginations into the PIVs generate SIVs (blue arrows), which directly receive mother cell cytoplasm. Upon burst of the mother cell, the SIVs are released. Alternatively, during growth in liquid media, mostly extracellular EVs are produced (red arrows), which appear transiently connected to the donor cell by thin strands of membrane material, which result in formation of string-like cell assemblies (pearling phenotype), which may still exchange cytoplasmic components.

In conclusion, the data presented here demonstrate that two L-form proliferation mechanisms, that is, generation of external as well as internal progeny, can occur in the same bacterial strain (see model in Fig. 6). The two proliferation modes do not seem to be mutually exclusive, and may be affected by various culture conditions.

Likely due to its relatively uncoordinated nature, the efficiency of progeny formation is much lower in L-forms compared to their walled counterparts. In this work we identified several characteristics that support L-form multiplication, such as the polyploidy and interconnectivity during growth. Yet, although the available evidence argues for it, it is still not clear whether L-form replication is an entirely passive process similar to what has been described for giant unilamellar vesicles (GUVs^30^), or if energy-dependent cellular mechanism(s) may be involved. Many of the features displayed by viable L-form cells resemble basic properties of abiotic, artificial lipid membrane vesicles^10^. In GUVs, a variety of proliferation modes similar to those observed for L-forms can be specifically triggered by variation of temperature^31^,^32^,^33^, pH^34^, osmolarity^35^, or exposure to detergents^36^ and other substances featuring extended hydrocarbon chains^28^,^37^. Similar as for L-forms, the underlying mechanism that drives generation of new GUV ‘progeny' appears to be an increasing surface-to-volume ratio, which is less stable and thermodynamically unfavourable, and is immediately reduced following fragmentation into smaller vesicles^38^. However, for complete membrane fission to occur between connected lipid vesicles, a significant energy threshold needs to be overcome^39^. In absence of this, the process often results in generation of beads-on-a-string-like assemblies^40^,^41^,^42^, which resemble the structures described here (Figs 1c,d,f and 5a–c and Supplementary Fig. 1). Upon exposure of growing *Listeria* L-form cultures to physical shear forces (such as vigorous swirling and shaking), the strands can break and individual cells are set free (data not shown). Similarly, abiotic lipid vesicles can also be released by applying mild shear forces, whereby encapsulated RNA, representing a model for a primitive genome, is randomly distributed to the newly formed vesicles^43^. Furthermore, it was demonstrated that larger GUVs containing DNA can undergo spontaneous division upon exposure to membrane precursors, whereby the encapsulated DNA is also distributed into smaller progeny vesicles^44^. Remarkably, self-reproducing GUVs containing higher concentrations of DNA featured an elevated frequency of spontaneous division, compared to GUVs comprising less or no DNA. Based on these findings, it may be hypothesized that high density of chromosomal DNA in enlarged L-form cells may contribute to the initiation of membrane perturbations and shape changes resulting in distribution of one or more genome copies into newly formed vesicles.

Altogether, the fascinating analogies between reproductive mechanisms of viable L-form cells and abiotic giant lipid vesicles support the hypothesis that L-form replication relies on non-dedicated mechanisms, perhaps resembling a very primitive and simplified state of cell multiplication. L-forms constitute a unique model for the study of potential mechanisms of growth and reproduction of hypothetical primordial cells^10^,^14^ in the absence of the highly complex set of structural elements employed by the current bacterial cell division machinery.

## Methods

### Bacterial strains and growth conditions

*Listeria monocytogenes* EGDe^45^ and a stable L-form variant^17^ derived from it were used in all experiments. The walled cells were grown in BHI medium at 30 °C, the L-forms in LLM at 32 °C, if not stated differently. *E. coli* XL1-Blue MRF′ was used for cloning and was cultivated in LB medium at 37 °C. All strains used in this study are listed in Supplementary Table 1.

### L-form induction and growth

The L-form was generated as described previously^15^, but in addition adapted to grow in liquid LLM medium after being cultivated in LLM soft agar. Briefly, an overnight culture of walled *L. monocytogenes* EGDe was inoculated into LLM soft agar (37 g l^−1^ BHI (Biolife, Italy), 150 g l^−1^ sucrose, 2.5 g l^−1^ MgSO_4_ × 7 H_2_O, 3 g l^−1^ milk serum powder (Emmi, Switzerland), 3 g l^−1^ agar) supplemented with 50 μg ml^−1^ Penicillin G (Penicillin G sodium salt, Sigma-Aldrich, USA). After several days, colonies were isolated and further cultivated in LLM soft agar with Penicillin G. These protoplast-like transient L-forms were stabilized by gradually reducing the Penicillin G concentration of the soft agar (50, 25 and 12.5 μg ml^−1^) during passaging. After continuous subcultivation in LLM soft agar in the absence of Penicillin G, an LLM liquid culture was inoculated with colony material obtained from soft agar. Growth could be observed after 1–2 weeks. After repeated passaging in order to adapt the L-forms to the liquid medium, a glycerol stock was prepared from this culture. From the time point where L-forms were grown in liquid culture, the 3 g l^−1^ of milk serum powder in the LLM was replaced with 1% heat-inactivated horse serum (Sigma-Aldrich, USA), and all experiments were performed with the new LLM formulation (37 g l^−1^ BHI, 150 g l^−1^ sucrose, 2.5 g l^−1^ MgSO_4_ × 7 H_2_O, 1% heat-inactivated horse serum). L-forms were cultivated in LLM at 32 °C, without shaking.

### Microscopy

Microscopy, except for the FLIP experiments (see below), was performed on a Leica TCS SPE confocal system (Leica Microsystems GmbH, Germany) equipped with a HCX PL FLUOTAR 100.0 × 1.30 oil objective. The following dyes were used: 40–50 μg ml^−1^ FM 4–64 (Life Technologies, USA), 10–100 μg ml^−1^ Hoechst 33342 (Life Technologies, USA), 50 μM CellTrace BODIPY TR methyl ester (BTME, Life Technologies, USA). The cells were mixed with the dyes and observed following an incubation time of 5 min. GFP was excited at a wavelength of 488 nm, the membrane dyes BTME and FM 4–64 at 532 nm. Micrograph images were analysed and prepared for publication using ImageJ Software (Version 1.48e, National Institutes of Health, USA).

### Time-lapse analysis of L-form proliferation

L-form liquid cultures (1–3-day-old) were vortexed and diluted 1:100 in LLM. A volume of 500 μl was transferred into a glass bottom dish (μ-Slide 2-well glass bottom, IBIDI, Germany). After careful slow centrifugation at 250*g* for 5 min, the supernatant was discarded and the attached cells overlaid with 1 ml of approximately 37 °C warm LLM soft agar. After the soft agar solidified, the dish was sealed with Parafilm in order to minimize evaporation of the medium. Time-lapse images were recorded during incubation in a heated microscope chamber at 32 °C, using a Leica TCS SPE confocal microscope.

### L-form transformation

Initially, transformation of L-form cells was done using a PEG-based method^25^. Briefly, 100 μl of a 2–3-day-old L-form culture was mixed with 20 μl of plasmid and 150 μl of PEG-8000 solution (3 g PEG-8000 in 10 ml LLM, filter-sterilized). After gentle mixing, the solution was incubated for 10 min on ice, followed by 10 min at 32 °C. Subsequently, 1 ml of LLM was added, the cells were incubated for 4 h at 32 °C, and plated on LLM agar supplemented with appropriate antibiotics.

Later in our study we developed a protocol for higher transformation efficiency using electroporation. For this, 4 ml of a 2–3-day-old L-form culture was cooled on ice and washed two times (5,000*g*, 5 min, 4 °C) with chilled sucrose-glycerol washing buffer (10% glycerol, 500 mM sucrose; pH adjusted to 7 with 100 mM NaOH; filter sterilized)^46^. The final L-form cell pellet was resuspended in 50 μl sucrose-glycerol washing buffer, and mixed with 10 μl of plasmid. After electroporation at 10 kV cm^−1^ (400 Ω, and 25 μF), 1 ml of LLM was added and the cells were recovered for 4 h at 32 °C before being plated on LLM agar supplemented with appropriate antibiotics.

### Generation of L-forms expressing fluorescent proteins

The GFP expressing L-form strain was generated by transformation with the integrative plasmid pPL3e/*gfp*^47^, where GFP expression is under the control of the constitutive *pHyper* promoter.

For RFP expression, a pPL2 (ref. 22) derivative which contains a *Listeria* codon-optimized *tagRFP* (*Lm-RFP*) under the control of a strong constitutive promoter (P_xyl/tetO_, Sibylle Schmitter, ETH Zurich, unpublished) was constructed. Briefly, purified pPL2, with a constitutive promoter between the SacI and EagI restriction sites, was digested with PstI and SalI. The *Lm-RFP* gene was amplified with primers PstI_RBS_LmRFP_F and SalI_Stop_LmRFP_R, digested with PstI and SalI, and ligated into the vector, yielding pPL2/*rfp.*

To express FtsZ-GFP in *Listeria*, we placed FtsZ-GFP under control of the inducible rhamnose promoter^19^ in two different plasmids, the cytoplasmic pAUL-A^20^ and the integrative pPL2 (ref. 22). For pAUL-A/*P*_*Rha*_*-ftsZ-gfp*, pAUL-A/*P*_*Rha*_was linearized by digestion with KpnI and dephosphorylation. *ftsZ* was amplified using primers ftsZ_FkpnI and ftsZ_R, and *gfp* was amplified using primers gfp_F and gfp_RkpnI. All three linear constructs contained complementary sequences and were assembled using the Gibson method^48^. For pPL2/*P*_*Rha*_*-ftsZ-gfp,* pPL2 was linearized by digestion with BamHI and subsequent dephosphorylation. *P*_*Rha*_*-ftsZ-gfp* was amplified from the pAUL-A/*P*_*Rha*_*-ftsZ-gfp* construct using primers PRha(pPL2)_F_inv and gfp_Rinverted. This digested product was then ligated into linearized pPL2 using Gibson assembly. Primer sequences are listed in Supplementary Table 2.

### Effect of the FtsZ inhibitor PC190723

A stock was prepared with a concentration of 10 mg ml^−1^ PC190723 (Calbiochem, Merck Millipore, Germany) in DMSO. In the first experiment, an overnight culture of walled *L. monocytogenes* and 1-day-old L-form culture were diluted 1: 10,000 in LLM supplemented with 8 μg ml^−1^ PC190723, or just DMSO as control, and incubated without shaking at 32 °C. For L-forms, we in addition tested 32 μg ml^−1^ PC190723. For enumeration, the cultures were spotted on LLM agar after 0, 8, 24, 32 and 48 h. In the second experiment, the cultures were initially supplemented with 16 μg ml^−1^ PC190723 or DMSO as a control, and then the same amount of PC190723 or DMSO was added after 12, 24, 36 and 48 h. After spotting, all the plates were incubated at 32 °C. Walled cells were enumerated after 48 h, whereas L-form cells were enumerated after 14 days. The agar plates with L-forms were sealed with Parafilm to prevent them from drying out. Three independent cultures were tested per cell type/condition (biological triplicates) and every culture was plated in duplicate (technical duplicates). The mean±standard deviation was calculated based on the biological triplicate.

To determine the effect of PC190723 on bacterial morphology, walled *L. monocytogenes* and 1-day-old L-forms were diluted 1:20 in LLM supplemented with 16 μg ml^−1^ PC190723 or an equivalent amount of DMSO as control and observed microscopically after 16 h incubation.

To determine the effect of PC190723 on FtsZ-GFP localization, 1 ml of a 2-day-old FtsZ-GFP L-form culture was vortexed, split in two 0.5 ml aliquots and either supplemented with 16 μg ml^−1^ PC190723 or an equivalent amount of DMSO. After 7.5 h at 32 °C, L-forms from both cultures were harvested and imaged by confocal fluorescence microscopy, with the same settings (excitation wavelength: 488 nm, emission wavelength: 490–637 nm). The ROI were chosen according to the cell boundaries observed in the phase contrast channel and histograms were generated with the software ImageJ. The normalized histograms of ten cells were averaged for each sample for quantitative analysis of the degree of FtsZ-GFP polymerization.

### Micromanipulation of single cells

A micromanipulator system consisting of two TransferMan NK2 devices (Vaudaux-Eppendorf, Germany) in combination with a CellTram Air (Vaudaux-Eppendorf, Germany) was installed on the inverted Leica TCS SPE microscope. In order to isolate single cells, 80 μl of diluted liquid culture was put on a cover slip and placed on the stage of microscope. The CellTram Air was used equipped with a 6 μm capillary to aspirate individual cells. To assure the capturing of only a single L-form, the air inside the capillary was first pushed to the front of the capillary to exclude trapping of unwanted structures. Captured cells were first transferred into a sterile tube containing 100 μl LLM broth, which was then inoculated into fresh media. Isolation of SIVs was performed as follows: after capture of a single L-form containing SIVs with a TransferMan capillary, a second TransferMan device equipped with a 0.4 μl microcapillary pipette was applied in order to puncture and mechanically disrupt the cell membrane of the L-form. As a result, internal vesicles were released into the wider 6 μm capillary, immediately followed by careful transfer into fresh medium and incubation at 32 °C for up to 16 days. If a new colony started to grow, the cells were inspected by microscopy to confirm GFP fluorescence and hence their origin.

### Quantification of L-form cell growth

A 1-day-old L-form culture was diluted 1:10,000 in LLM, and 1 ml cultures were prepared in 2 ml Eppendorf tubes. After 0, 1, 2, 3, 7 and 14 days, L-form growth was monitored by plating on LLM agar, optical density measurement and determination of chromosome numbers. For evaluation of colony-forming units the agar plates were sealed with parafilm to prevent them from drying out and they were incubated for 14 days at 32 °C. For chromosome number determination, 25 μl of a homogenized L-form culture was mixed with 25 μl of 2% Triton X-100, and incubated at 95 °C for 30 min, with vortexing every 10 min. This extract was then diluted 1:100 with deionized water (Milli-Q) to minimize inhibitory effects during PCR. Quantitative qPCR was performed using the Rotor-Gene 6,000 cycler (Corbett Life Science), and the KAPA SYBR FAST Universal qPCR Kit (Kapa Biosystems), according to the manufacturer's instructions. The single copy *rpoB* gene was used as reference and standard to determine chromosome numbers. The primers for determination of the *rpoB* copy numbers were rpoB_F and rpoB_R. A DNA standard with known copy numbers prepared from *L. monocytogenes* EGDe chromosomal DNA was used to quantify the absolute copy numbers for the investigated culture aliquots. For every time point and method, three individual 1 ml cultures (biological triplicates) were measured and all cultures were measured in duplicates (technical duplicates). The mean±standard deviation was calculated based on the biological triplicate. Primer sequences are listed in Supplementary Table 2.

### Chromosome quantification by Hoechst 33342 quantification

Walled and L-form cells were harvested during exponential growth in LLM, and fixed by mixing 100 μl of culture with 100 μl of 8% formaldehyde in 0.5 M sucrose, followed by incubation for 1.75 h at room temperature. Subsequently, 2 μl of Hoechst 33342 (1 mg ml^−1^) was added and the cells were incubated at least 30 min before being imaged on LLM containing agar pads with the Leica TCS SPE (excitation at 405 nm, emission at 412–526 nm). Three independent cultures were imaged for both, L-form and walled cells, and analysed as follows: The DNA content of each cell was estimated as the product of the intensity and the volume of the Hoechst 33342 signal, as described in Supplementary Fig. 4. Contour determination of the Hoechst 33342 signal for intensity measurement and volume estimation was performed using the following ImageJ software macro, for images where the first channel is the fluorescent Hoechst 33342 channel:

run(‘Duplicate...', ‘duplicate channels=1');

run(‘Gaussian Blur...', ‘sigma=2');

setAutoThreshold(‘Otsu');

setOption(‘BlackBackground', false);

run(‘Convert to Mask');

run(‘Invert');

run(‘Watershed');

run(‘Analyze Particles...', ‘size=0.20-Infinity show=Nothing exclude clear add');

In a few cases when the software did not properly recognize L-forms emitting only weak signals, the contour was corrected manually (arrows in Supplementary Fig. 4a). Three independent cultures were measured for each cell type (biological triplicate), and at least 50 cells per culture and cell type were analysed. The mean±standard deviation was calculated based on the biological triplicate.

### Labelling of DNA replication sites

In order to monitor the DNA replication processes occurring in L-forms, chemical labelling of modified nucleosides freshly incorporated into new DNA was used (Click-iT EdU Alexa Fluor 488 Imaging Kit, Thermo Fisher Scientific, USA) was used. For this, 195 μl of actively growing, 2-day-old L-forms were mixed with 5 μl EdU stock solution (10 mM). The cells were incubated for 1 h at 32 °C and subsequently fixed by addition of 200 μl 8% formaldehyde in 0.5 M sucrose, and incubation for 15 min at room temperature. Subsequently, 20 μl 10% Triton X-100 was added, and the cells were incubated for 15 min at room temperature for permeabilization. Then, 600 μl of 0.5 M sucrose was added, the cells were centrifuged at 1,000*g* for 5 min, and the supernatant was removed. The pellet was subsequently treated with 100 μl Click-iT reaction cocktail for 30 min at room temperature, followed by washing with 0.5 M sucrose (1,000*g*, 5 min), and 30 min of incubation with Hoechst 33342. Finally, the cells were again washed with 0.5 M sucrose and resuspended in 50 μl 0.5 M sucrose, before being inspected by confocal fluorescence microscopy (Leica TCS SPE, excitation wavelength: 488 nm for EdU-Alexa Fluor 488, 405 nm for Hoechst 33342).

### Shear stress stability of L-form lipid strands

To determine the mechanical stability of the L-form connecting lipid strands, L-forms were exposed to different flow rates in a microfluidic flow cell (μ-Slide VI^0.4^, IBIDI) using a MINIPULS Evolution peristaltic pump (Gilson, USA) equipped with a MF4 pump head and platinum-cured silicone tubing with and internal diameter of 1.8 mm. L-form cells were introduced into the channel of the flow cell via the peristaltic pump by aspirating a few μl of a 1-day-old L-form liquid culture, followed by aspiration of sterile LLM. The system was run with a flow rate of 570 μl min^−1^ until the cells reached the flow channel. Then, the flow was stopped and the cells were allowed to adhere for 2 h. Subsequently, the system was run at flow rates from 90 to 300 μl min^−1^ to flush away the non-attached L-forms for several minutes. The remaining L-forms were subjected to variable flow rates ranging from 30 to 966 μl min^−1^, while being recorded using the Leica Application Suite Software (Version 2.5.1.6757, Leica, Germany) on a Leica TCS SPE microscope. During image recording, 2 × 2 binning was enabled to allow a higher frame rate.

### FLIP experiments

FLIP experiments were performed on a Leica TCS SP5 system, equipped with a HCX PL APO lambda blue 63.0 × 1.40 OIL UV objective (Center for Microscopy and Image Analysis, University of Zurich). A volume of 4–5 μl of 1–day-old GFP expressing L-forms were spotted onto a microscope slide, and a glass cover slip was put on top. In certain cases, the L-forms were spotted onto an agar pad containing 500 mM sucrose or they were added onto a glass bottom dish (μ-Slide 2 well glass bottom, IBIDI, Germany), and a small piece of LLM agar was placed on top in order to minimize uncontrolled motion of the cells. Excitation and bleaching of the fluorophores was performed using a wavelength of 488 nm. The resolution of the photomultiplier was 12 bits, and laser intensity during measurement was adjusted to a value where no oversaturated pixels were detected. A total of 2–10 bleach cycles were performed with laser intensity set to 80–100%. The DIC (differential interference contrast) channel was monitored in parallel with the fluorescence, to make sure that the focal plane stayed constant during the experiment.

### Data availability

The authors declare that the data supporting the findings of this study are available within the article and its Supplementary Information files, or from the corresponding author on request.

## Additional information

**How to cite this article:** Studer, P., *et al*. Proliferation of *Listeria monocytogenes* L-form cells by formation of internal and external vesicles. *Nat. Commun.* 7:13631 doi: 10.1038/ncomms13631 (2016).

**Publisher's note**: Springer Nature remains neutral with regard to jurisdictional claims in published maps and institutional affiliations.

## Supplementary Material

Supplementary Information Supplementary Figures 1-7, Supplementary Tables 1-2 and Supplementary References

Supplementary Movie 1 A *Listeria monocytogenes* L-form cell proliferating by a budding-like process.

Supplementary Movie 2 An L-form cell forming multiple protrusions that finally pinch off, resulting in the formation of new L-form cells.

Supplementary Movie 3 Two L-form cells, which are connected via thin strands, form massive new strand material and associated new L-form bodies. The strands move quickly due to Brownian motion.

Supplementary Movie 4 An L-form cell producing internal primary (PIV) and secondary (SIV) vesicles.

Supplementary Movie 5 FtsZ-GFP can form filaments throughout the L-form cytoplasm. 3D reconstruction from confocal z-stack images. Green: FtsZ-GFP, Red: Membrane stain BTME

Supplementary Movie 6 L-form strands display high mechanical stability and flexibility. The connecting strands of L-forms are stretched when they are subjected to high flow rates in a flow chamber and they contract again when the flow rate is reduced.

Supplementary Movie 7 The cytoplasm of two L-form cells which are connected via two small L-form bodies forms a continuum as shown by the loss of fluorescence in the "connected cell" upon bleaching of the "bleached cell". The fluorescence intensity profiles of the three marked cells in the movie can be found in Supplementary Figure 7.
