# Supplementary material for: Proliferation of Listeria monocytogenes L-form cells by formation of internal and external vesicles
Source: Nat Commun. 2016 Nov 23;7:13631. doi: 10.1038/ncomms13631 (PMC5123018; doi:10.1038/ncomms13631)
Supplement: Supplementary Information — Supplementary Figures 1-7, Supplementary Tables 1-2 and Supplementary References [file ncomms13631-s1.pdf]

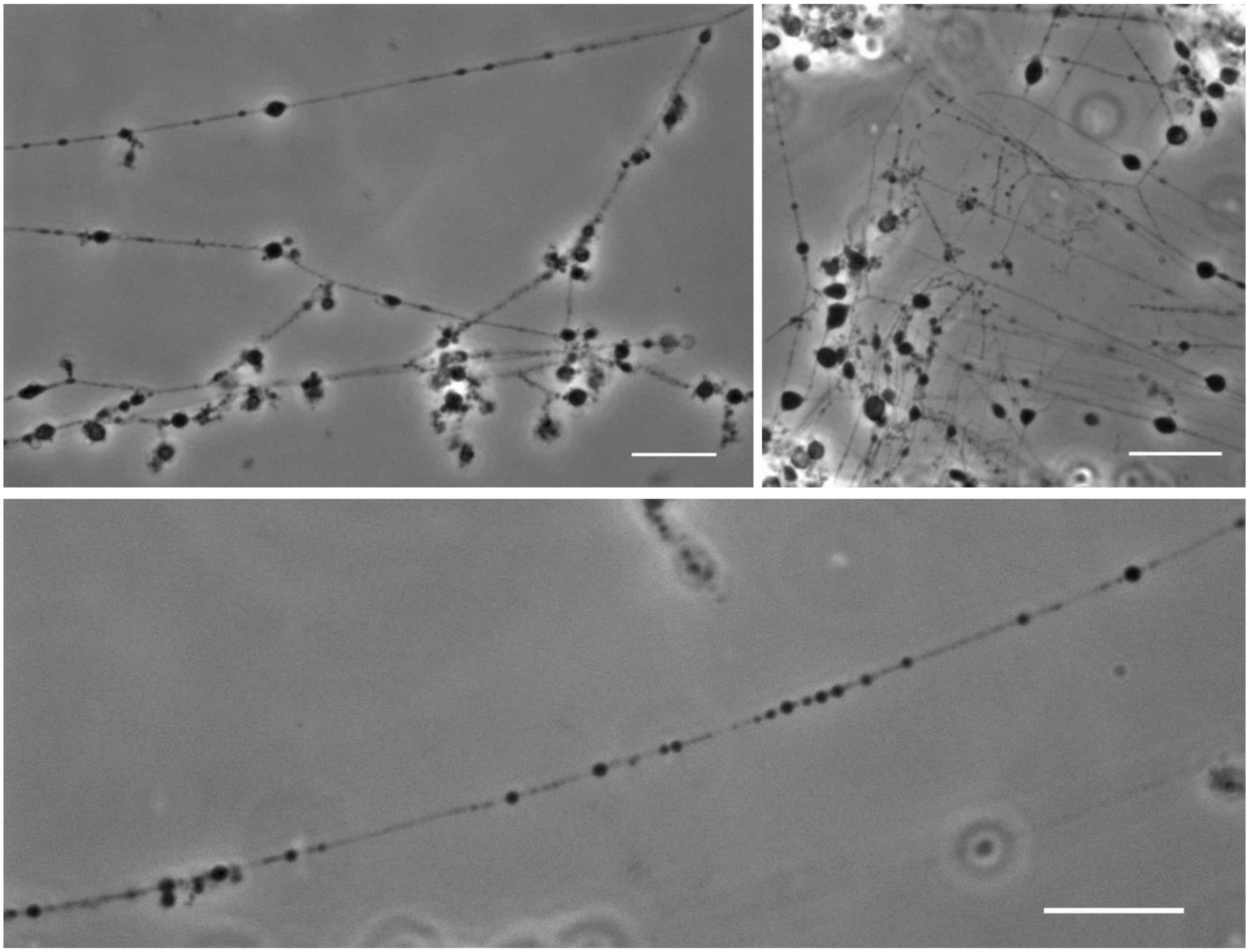

Supplementary Figure 1: Representative images of L-forms grown in liquid culture. The individual L-form cells remained connected and feature cytoplasmic continuity during the propagation process. Scale bar, 10 µm

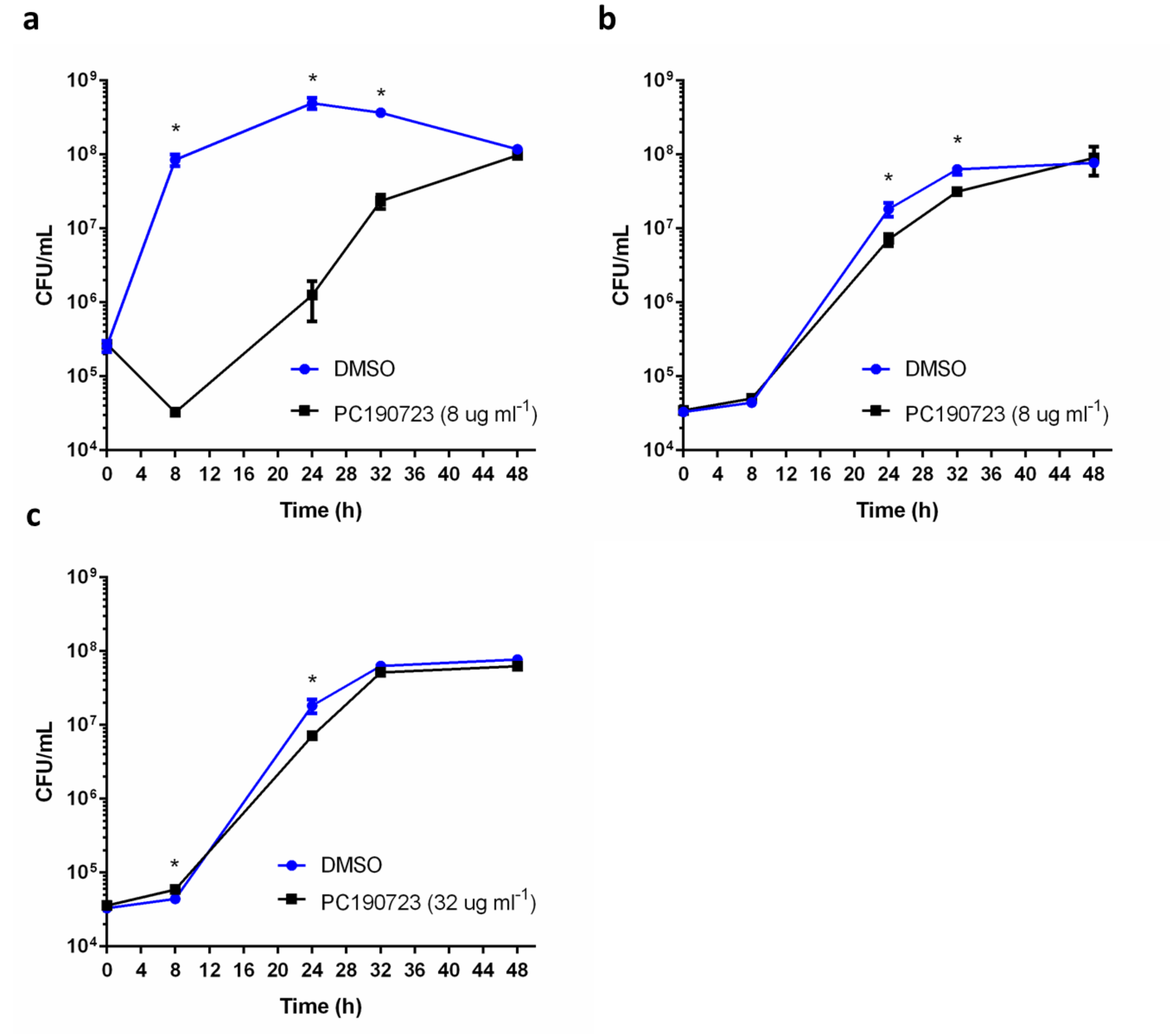

Supplementary Figure 2: Effect of PC19023 on bacterial growth. (a) Walled cells displayed a severe growth delay in presence of 8 µg ml^{-1} PC190723, but resumed growth after approximately 8 h, probably due to inhibitor degradation. (b) L-form cells showed a comparable growth rate in absence of PC190723 and in presence of 8 µg ml^{-1} PC190723 (b) and 32 µg ml^{-1} PC190723 (c). Values represent average ± s.d. of three independent cultures (n = 3). Asterisks indicate $P < 0.05$, based on an unpaired *t* test.

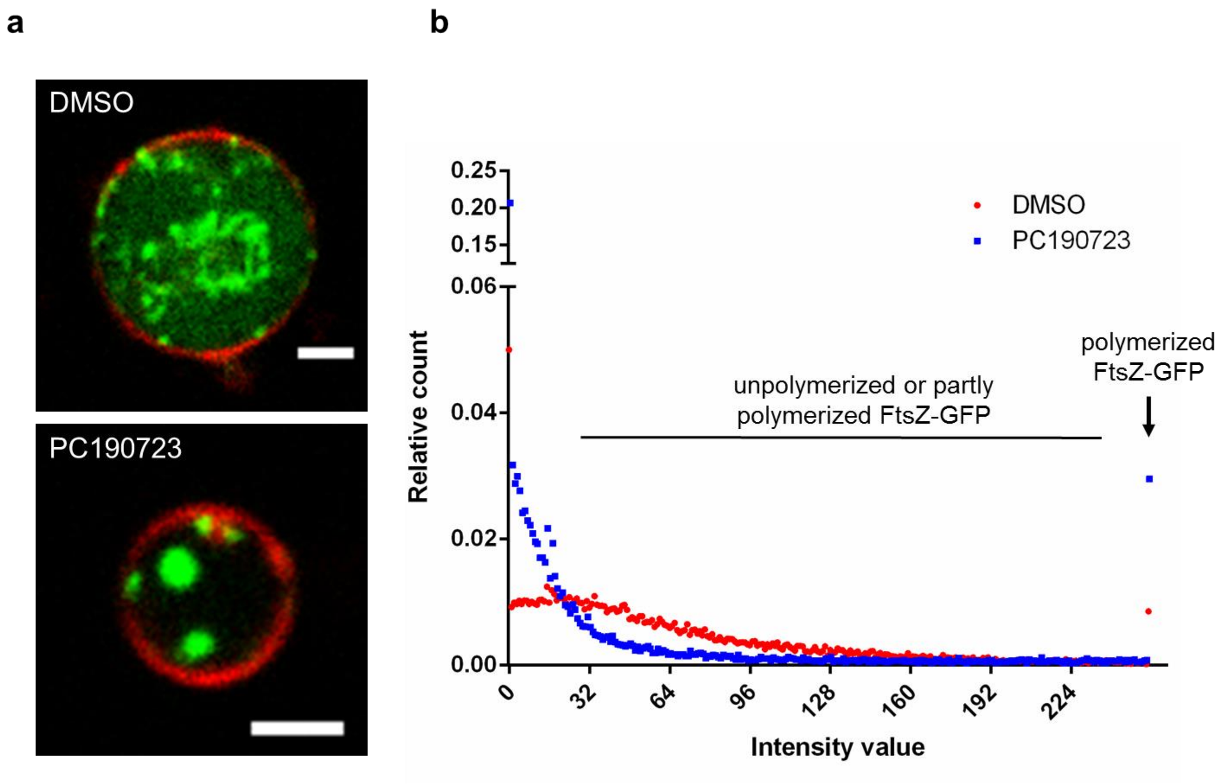

Supplementary Figure 3: PC190723 specifically affects FtsZ function in *Listeria monocytogenes*. (a) Stable L-form cells derived from L. monocytogenes EGDe constitutively expressing FtsZ-GFP from episomal pAUL-A plasmid were used to verify the stabilizing effect of PC190723 on FtsZ filaments. PC190723 reduced the relative amount of unpolymerized FtsZ-GFP molecules (weak dispersed fluorescence signal), while it enhanced the relative amount of polymerized FtsZ-GFP (distinct fluorescent foci). The cells were stained with the membrane dye FM4-64 (red). Scale bar, 2 µm. (b) The graph shows the normalized histogram of the FtsZ-GFP signal averaged over ten PC190723 treated and DMSO (control) treated cells. Each dot represents the relative abundance of pixels with the respective intensity value. Higher intensity corresponds to a higher degree of FtsZ-GFP polymerization, due to the close spatial accumulation of multiple FtsZ-GFP molecules. Fully polymerized FtsZ-GFP has a maximum intensity value of 255. The histogram of PC190723 treated cells shows more polymerized FtsZ-GFP (arrow at maxima with intensity value of 255), and less amounts of unpolymerized FtsZ-GFP compared to DMSO treated cells.

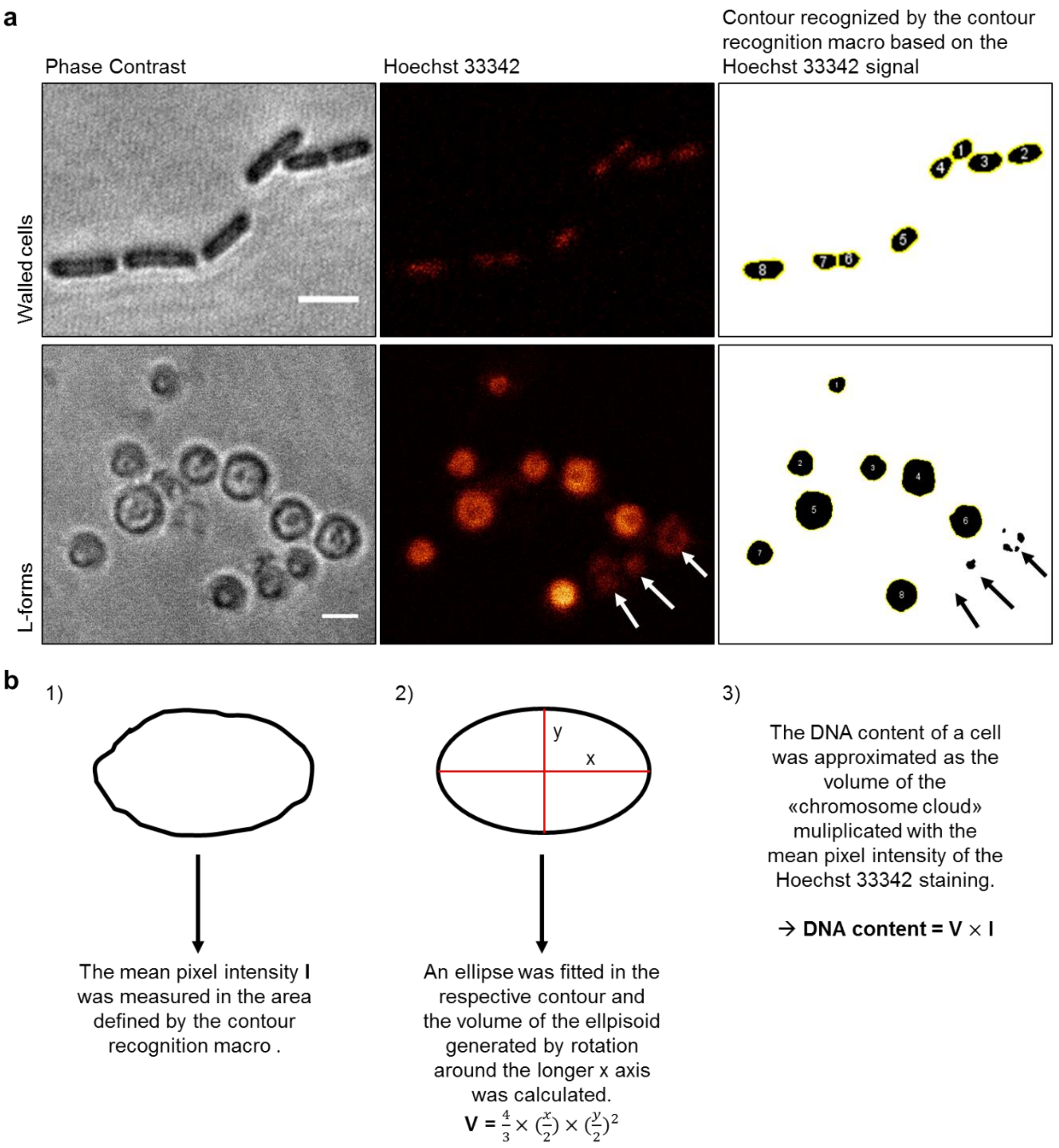

Supplementary Figure 4: Quantification of DNA content. (a) Representative images of walled and L-form cells exposed to the DNA-specific dye Hoechst 33342, and the respective contour image signals produced by the ImageJ macro. Note that L-forms with a weak signal were sometimes not recognized by the ImageJ macro (arrows). In such cases, the cell boundaries were drawn manually. Scale bar, 2 µm. (b) Description of the approach applied to determine the DNA content per cell.

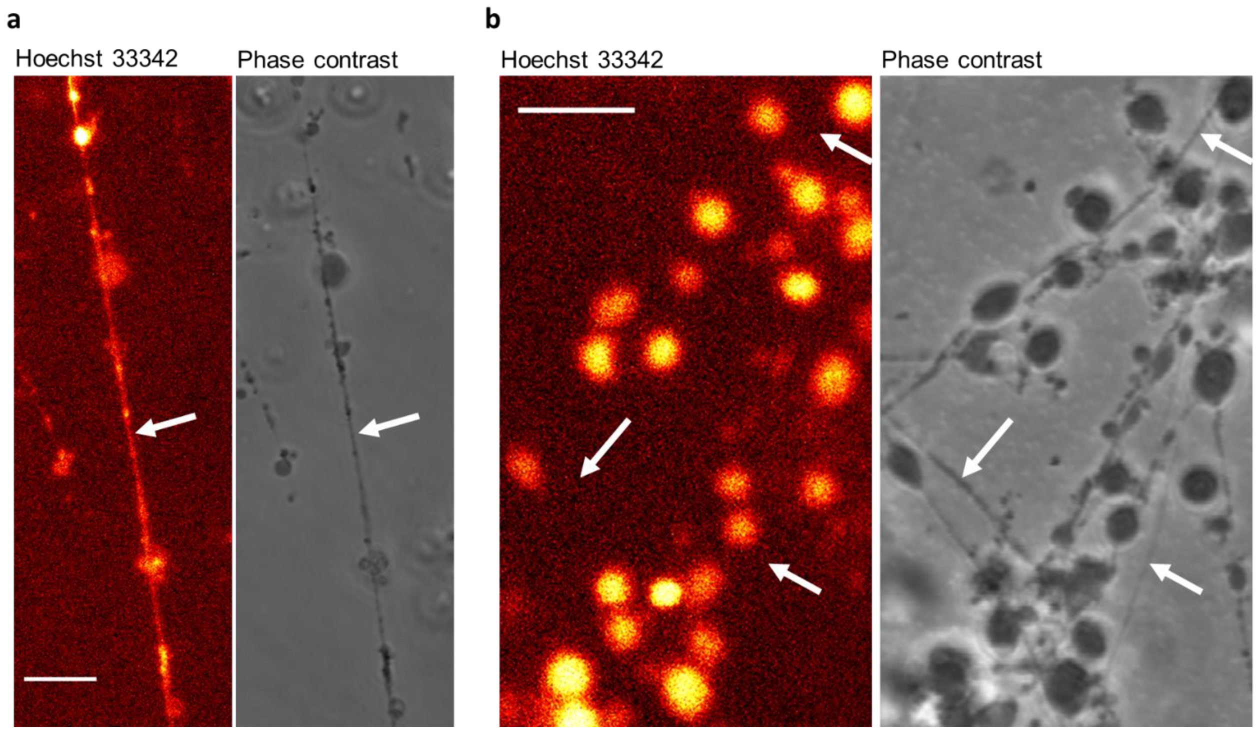

Supplementary Figure 5: Hoechst 33342 staining of L-forms featuring strands. (a) Some strands are filled with DNA as indicated by the fluorescent signal (arrow), while others (b) appear to lack DNA as indicated by the lack of fluorescence in the strands (arrows). Scale bars, 5 µm.

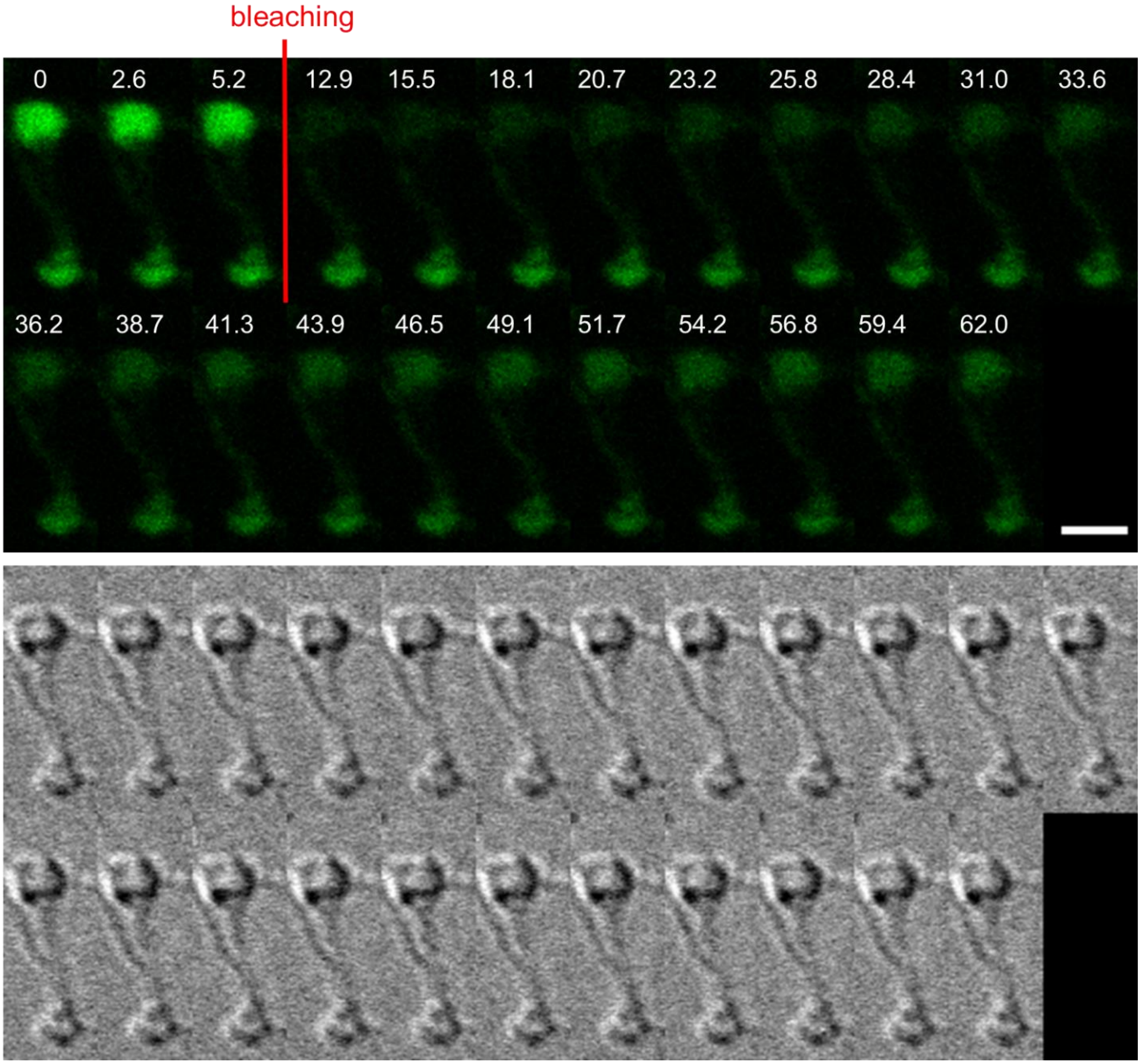

Supplementary Figure 6: Assembly of the individual time-lapse frames from a FLIP experiment on two connected L-form cells. Recording the fluorescence intensity of the GFP molecules of two connected cells for approx. 1 min after bleaching the upper cell (top panel) revealed reconstitution of the fluorescence in the bleached cell, while the fluorescence in the second, non-bleached cell is decreasing. The corresponding images from the DIC channel (lower panel) indicate that the cells did not move during the time course of the experiment. Scale bar, 2 µm. Note that during photobleaching, no images can be recorded.

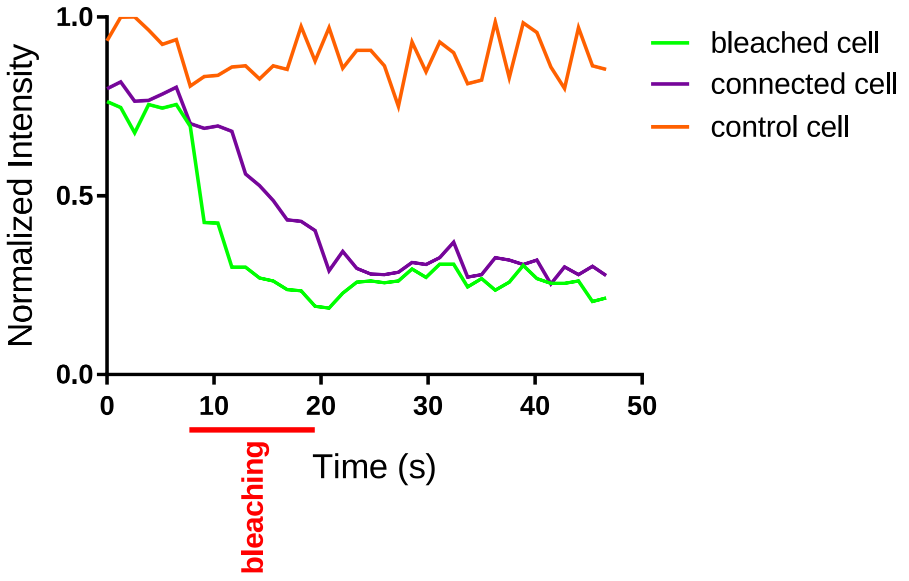

Supplementary Figure 7: Normalized fluorescence intensity of the cells featured in Supplementary Movie 7. Initially**,** fluorescence intensity of the bleached cell drops, followed by the fluorescence signal of the connected cell, with a delay of a few seconds. The fluorescence intensity of an unconnected control cell remained constant.

Supplementary Table 1: Bacterial strains used in this study.

Strain	Comments	Source
L. monocytogenes EGDe	Walled *Listeria* strain that was used for L-form induction	Glaser *et al*. [1]
L. monocytogenes EGDe^{sL}	Stable L-form derivative of EGDe	Studer *et al.* [2]
L. monocytogenes EGDe^{sL}::pPL3e/*gfp*	Stable L-form constitutively expressing cytosolic GFP	This work.
L. monocytogenes EGDe^{sL}::pPL2/*rfp*	Stable L-form constitutively expressing cytosolic TagRFP	This work.
L. monocytogenes EGDe::pPL2/P_{Rha}-*ftsZ-gfp*	Walled *Listeria* strain expressing FtsZ-GFP (low-level expression, Rhamose-inducible expression)	This work.
L. monocytogenes EGDe pAUL-A/P_{Rha}-*ftsZ-gfp*	Walled *Listeria* strain expressing FtsZ-GFP (high-level expression, constitutive expression)	This work.
L. monocytogenes EGDe^{sL}::pPL2/P_{Rha}-*ftsZ-gfp*	Stable L-form expressing FtsZ-GFP (low-level expression, Rhamose-inducible expression)	This work.
L. monocytogenes EGDe^{sL} pAUL-A/P_{Rha}-*ftsZ-gfp*	Stable L-form expressing FtsZ-GFP (high-level expression, constitutive expression)	This work.
E. coli XL1-blue MRF’	Used for cloning	Stratagene California, La Jolla, California, USA
E. coli XL1-blue MRF’ pPL3e/*gfp*	Used for prepping pPL3e/*gfp*	This work.
E. coli XL1-blue MRF’ pPL2/*rfp*	Used for prepping pPL2/*rfp*	This work.
E. coli XL1-blue MRF’ pPL2/P_{Rha}-*ftsZ-gfp*	Used for prepping pPL2/P_{Rha}-*ftsZ-gfp*	This work.
E. coli XL1-blue MRF’ pAUL-A/P_{Rha}-*ftsZ-gfp*	Used for prepping pAUL-A/P_{Rha}-*ftsZ-gfp*	This work.

Supplementary Table 2: Primers used in this study.

Primer name	Sequence (5'-3')
rpoB_F	CGC GAA TCA GTG AAG TAC TTG
rpoB_R	ATC CTC AAT TGG CGA AAT ATC
PstI_RBS_LmRFP_F	TTT CTG CAG AGG AGG GAA TCG ATA TGG TTG
SalI_Stop_LmRFP_R	TTT GTC GAC TTA ATT AAG TTT ATG GCC TAA TTT G
ftsZ_FkpnI	CTT AAC TAA AAT GAG TGG TAG GAG GCA ATA ATA TGT TAG
ftsZ_R	AAA AGT TCT TCT CCT TTA CTC ATT CCG CGA CGG TTA CGG
gfp_F	GTA ACC GTA ACC GTC GCG GAA TGA GTA AAG GAG AAG AAC
gfp_RkpnI	TCT AGA GGA TCC CCG GGT TTA TTT GTA TAG TTC ATC CAT
PRha(pPL2)_F_inv	AAT TCC TGC AGC CCG GGG GAT ATT CCG TGA TAA TTT GGT
gfp_Rinverted	CGC TCT AGA ACT AGT GGA TTA TTT GTA TAG TTC ATC CAT

Supplementary references

1. Glaser P*, et al.* Comparative genomics of Listeria species. *Science* **294**, 849-852 (2001).

2. Studer P*, et al.* The Absence of a Mature Cell Wall Sacculus in Stable Listeria monocytogenes L-Form Cells Is Independent of Peptidoglycan Synthesis. *PLoS One* **11**, e0154925 (2016).
